# Electroacupuncture for Gastrointestinal Function Recovery after Gynecological Surgery: A Systematic Review and Meta-Analysis

**DOI:** 10.1155/2021/8329366

**Published:** 2021-12-21

**Authors:** Xiang Gao, Yuzhuo Zhang, Yizhe Zhang, YuTzu Ku, Yi Guo

**Affiliations:** ^1^Shanxi Province Hospital of Traditional Chinese Medicine, Shanxi Provincial Institute of Traditional Chinese Medicine, Taiyuan 030012, China; ^2^Guangzhou University of Chinese Medicine, Guangzhou 510120, China

## Abstract

**Background:**

Evidence for the efficacy and safety of electroacupuncture (EA) on gastrointestinal function recovery after gynecological surgery is unclear.

**Objective:**

This meta-analysis aimed to evaluate the effects of EA on recovery of postoperative gastrointestinal function for patients receiving gynecological surgery. Data sources: PubMed, Cochrane Central Register of Controlled Trials (CINAHL), Embase, China National Knowledge Infrastructure (CNKI), Weipu (CQVIP), and Wanfang databases were systematically searched from the inception dates to May 30, 2020, for relevant randomized controlled trials (RCTs). Study selection: RCTs that evaluated EA for postoperative gastrointestinal function directly related to gynecological surgery in adults aged 18 years or over. Data extraction and synthesis: paired reviewer independently extracted the data and assessed study quality. Standardized mean differences (SMD) were calculated as the effect measure from a random effects model. Main outcomes and measures: time to first flatus (TFF), time to bowel sounds recovery (TBS), and time to first defecation (TFD) were recorded as primary outcomes; postoperative nausea and vomiting (PONV), motilin (MTL), gastrin (GAS), pH value of gastric mucosa (pHi), gastric mucosal partial pressure of carbon dioxide (PgCO2), vasoactive intestinal peptide (VIP), and adverse event were reported as secondary outcomes.

**Results:**

We included eighteen RCTs (1117 participants). Our findings suggested that compared to the control group (CG), electroacupuncture group (EG) showed significant effects on TFF (SMD = −0.98, 95% CI: [−1.28, −0.68], *P* < 0.00001, *I*^2^ = 69%), TBS (SMD = −0.98, 95% CI: [−1.84, −0.12], *P*=0.03, *I*^2^ = 92%), and TFD (SMD = −1.23, 95% CI: [−1.59, −0.88], *P* < 0.0001, *I*^2^ = 0%). Moreover, the incidence of PONV at postoperative 6 h (OR = 0.42, 95% CI: [0.27, 0.64], *P* < 0.0001, *I*^2^ = 0%) and 24 h (OR = 0.46, 95% CI: [0.32, 0.68], *P* < 0.0001, *I*^2^ = 0%) was lower in the EG than that in the CG, whereas no significant difference in ratio of PONV at postoperative 48 h (OR = 0.55, 95% CI: [0.20, 1.51], *P*=0.25, *I*^2^ = 0%) was detected between the two groups. Meanwhile, there was a significant effect in favor of EA on the level of MTL at postoperative 6 h (SMD = −0.93, 95% CI: [−1.36, −0.61], *P* < 0.0001, *I*^2^ = 21%), while no significant effect was observed at postoperative 24 h (SMD = −0.43, 95% CI: [−0.89, 0.02], *P*=0.06, *I*^2^ = 69%) in the EG when compared to the CG. Additionally, a large significant effect on decreasing PgCO_2_ was found in the EG in comparison to the CG, but no significant effect in favor of EA on GAS, VIP, or pHi was observed. It was reported that there was one participant with pain at the needling sites and bruising, and three participants withdrew because they were not intolerant to EA.

**Conclusions:**

EA could be a promising strategy for the prevention and treatment of gastrointestinal dysfunction after gynecological surgery, including shortening TFF and TFD, TBS, regulating MTL, and decreasing the ratio of PONV within postoperative 24h. The effects on MTL and PONV varied with different intervention points, and EA used at 30 min prior to surgery might be recommended. However, the evidence quality ranged from low to very low, and large-scale and high-quality RCTs were warranted.

## 1. Introduction

Postoperative recovery of gastrointestinal function was considered as one of the most important parts for the rehabilitation after surgery, which was a condition that mainly related to surgical stress, anesthesia regimen, surgical treatment, and postoperative analgesia method [1]. The short-term gastrointestinal dysfunction after surgery was referred to as postoperative ileus (POI), which could cause undesirable consequences, including abdominal distension, lack of flatus and defecation, and nausea and vomiting. As was reported, POI would influence postoperative experience, increase the length of hospital stay, and even raise the risk of morbidity and mortality, which not only strongly prevented rehabilitation after surgery, but also posed a substantial economic burden on family and society [2].

POI was frequently observed after gastroenterological surgery, and it was followed by gynecological surgery [3]. It was reported that the prevalence of POI ranged from 5% to 25% among patients undergoing gynecological surgery [4], and even the incidence was up to 50% in patients receiving surgical therapy for gynecological cancers [5]. In recent years, minimally invasive surgery, such as laparoscopy surgery, has become more commonly used for gynecologic indications [6]. However, the incidence of gastrointestinal dysfunction following surgery is not decreased [7]. Even though increasing studies focusing on improving recovery of postoperative gastrointestinal function have been conducted, strategies with satisfactory efficacy are still rarely reported, especially for gynecological population. Thus, apart from improving anesthesia regimen and surgical technique, so far it is still urgent and essential to develop an effective and safety method of promoting the return of gastrointestinal function for patients receiving gynecological surgery.

Acupuncture, as one of the conventional Chinese medical therapies, has been applied to promote gastrointestinal function for thousands of years [8]. Electroacupuncture (EA) is a modified technique involving traditional acupuncture and electrical stimulation to achieve a greater response, which has been proved to be a promising approach to reduce complications and accelerate rehabilitation after surgery in the field of orthopedic, abdominal, and gynecological diseases. Moreover, there are an increasing number of studies focusing on EA for treatment and prevention of POI. But the results concerning the efficacy of EA on POI are inconsistent [9, 10]. It was reported in the previous meta-analyses [11–14] that supported the benefits of EA/acupuncture to POI, but the efficacy of EA on POI for gynecological patients still lacked evidence basis. Gynecological surgery was unlike gastroenterological surgery or other surgery in terms of pathology and surgical methods, which might lead to difference outcomes of gastrointestinal function recovery after surgery. Consequently, the purpose of this study was to evaluate the safety and efficacy of EA on postoperative recovery of gastrointestinal function for the patients receiving gynecological surgery, which could provide new evidence for promoting rehabilitation after gynecological surgery.

## 2. Methods

This study was conducted according to the Preferred Reporting Items for Systematic Reviews and Meta-Analyses (PRISMA) guidelines [15]. Moreover, the protocol of this study was registered in PROSPERO, and the registration number is CRD42021260096. In the process of retrieval, we found that most of the literatures were published in Chinese, so we retrieved with increased numbers of Chinese database. However, due to the delay of preliminary preparation time, the deadline for literature retrieval was extended to May 30, 2021. We had submitted the amendments in the registration system.

### 2.1. Search Strategy

In order to identify relevant studies, we systematically search the electronic databases including PubMed, Cochrane Central Register of Controlled Trials (CINAHL), Embase, China National Knowledge Infrastructure (CNKI), Weipu (CQVIP), and Wanfang from inception to May 30, 2021. The search terms, such as “postoperative ileus,” “postoperative gastrointestinal motility disorder,” “postoperative gastrointestinal function recovery,” “postoperative gastrointestinal dysfunction,” “postoperative gastrointestinal function,” “postoperative nausea and vomiting,” and “electroacupuncture,” were used to search in each database without language or disease restrictions. The search strategy was described in Supplementary Figure 1.

### 2.2. Inclusion Criteria

We formulated inclusion and exclusion criteria based on PICOS (patients, intervention, comparator, outcomes, and study design) approach [16].

#### 2.2.1. Patients

- Subjects aged 18 or over
- Subjects with gynecological disease receiving surgical treatment

#### 2.2.2. Interventions

EA should be used in the experimental group in the included study.

#### 2.2.3. Comparators

EA versus (vs) other therapy, EA+ other therapy vs. other therapy, EA vs. nonintervention.

#### 2.2.4. Outcomes

The literature reporting more than or equal to one index for assessing gastrointestinal function after surgery would be included in this study. The outcomes were listed as follows:

 
*Primary Outcomes*
(i) Time to first flatus (TFF)
(ii) Time to bowel sounds recovery (TBS)
(iii) Time to first defecation (TFD)
 
*Secondary Outcomes*
(i) Postoperative nausea and vomiting (PONV)
(ii) Motilin (MTL)
(iii) Gastrin (GAS)
(iv) pH value of gastric mucosa (pHi)
(v) Gastric mucosal partial pressure of carbon dioxide (PgCO_2_)
(vi) Vasoactive intestinal peptide (VIP)
(vii) Adverse event

#### 2.2.5. Study Design

- Clinical randomized controlled trials (RCTs)
- Published in a peer-reviewed journal
- Language: Chinese or English

### 2.3. Exclusion Criteria

(1) Pregnancy, or participants receiving obstetric surgery; (2) manual acupuncture, acupoint massage, transcutaneous electrical nerve stimulation, transcutaneous electrical acupoints stimulation, and neuromuscular electrical stimulation; (3) repeated publications, conference abstracts, comments, protocol, meta-analysis, or reviews, or full-text unavailable articles.

### 2.4. Literature Screening

Paired investigators (X. Gao and Yu Z. Zhang) independently screened the retrieved studies. Firstly, all the studies were imported to EndNote X8 (Bld 10063) to remove duplicates. Secondly, the remaining studies were preliminarily selected by reading the titles and abstracts. Thirdly, the included studies were identified based on the inclusion and exclusion criteria by full-text reading.

### 2.5. Data Extraction

The relevant data were independently extracted from the included studies by the two reviewers, including study characteristics (e.g., author names, publication year, study design, and sample size), participant characteristics (e.g., age, year), intervention type, intervention characteristics (e.g., acupoints, model, and intensity), and outcomes. During the process of screening and data extraction, any discrepancies would be resolved by discussion, or consultation with a third reviewer (Y. Guo) until a consensus was reached.

### 2.6. Quality Assessment

In accordance with the Cochrane Collaboration's Risk of Bias tool V 2.0, the two reviewers assessed the quality of the included literatures from seven dimensions: bias arising from the randomisation process, bias from deviations from the intended interventions, bias from missing outcome data, bias due to measurement of the outcome, bias from the selection of the reported results, and overall risk of bias [16]. The risk of each item is divided into three levels: high, unclear, and low. Meanwhile, the two researchers evaluated the methodological quality of included studies according to the Physiotherapy Evidence Database (PEDro) scale [17], which includes 10 items for assessment of trial quality from various aspects, including randomisation procedure, concealed allocation, similar baseline, patients blinding, therapists blinding, assessors blinding, adequate follow-up (dropout rate <15%), intention to treat analysis, between-group statistical analysis, and point and variability measures; the total score ranged from 0 to 10. According to the total score, quality of study was categorized into three degrees, including high (10 ≥ PEDro score ≥ 6), fair (6 > PEDro score ≥ 4), and low (0 ≤ PEDro score ≤ 3) [18].

### 2.7. Statistical Analysis

We conducted the data analysis by using review manager (version 5.3, the Nordic Cochrane Centre, Copenhagen, Denmark) and Stata (version 13.0, the StataCorp LP, TX, USA). For continuous variables, standardized mean differences (SMDs) and 95% confidence intervals (95% CI) were calculated as the effect measure by using a random effects model, and standard mean effect sizes were divided to different categories, including no change (0), small effect (0.2), moderate effect (0.5), and large effect (0.8) [18]. Enumeration data was analyzed by using a random effects method and expressed as the odds ratios (OR) and 95% CI. All the corresponding meta-analysis results were illustrated by the forest map intuitively. Cochran's *Q*-test and *I*^2^ index were employed to estimate heterogeneity [19], and *I*^2^ statistic greater than 50% was considered as substantially heterogeneous. When a substantially heterogeneous was observed, subgroups analysis or sensitivity analyses were used to determine the risk factor resulted in the high heterogeneity. Grading of recommendations, assessment, development, and evaluation (GRADE) approach was utilized to evaluate the quality of evidence [20]. In addition, publication bias was assessed by Begg's and Egger's tests [19], and the visual inspection of funnel plots would be applied if the index was reported in more than 10 included studies. The significant difference level was set at *P* < 0.05.

## 3. Results

### 3.1. Study Selection

**Figure 1:**
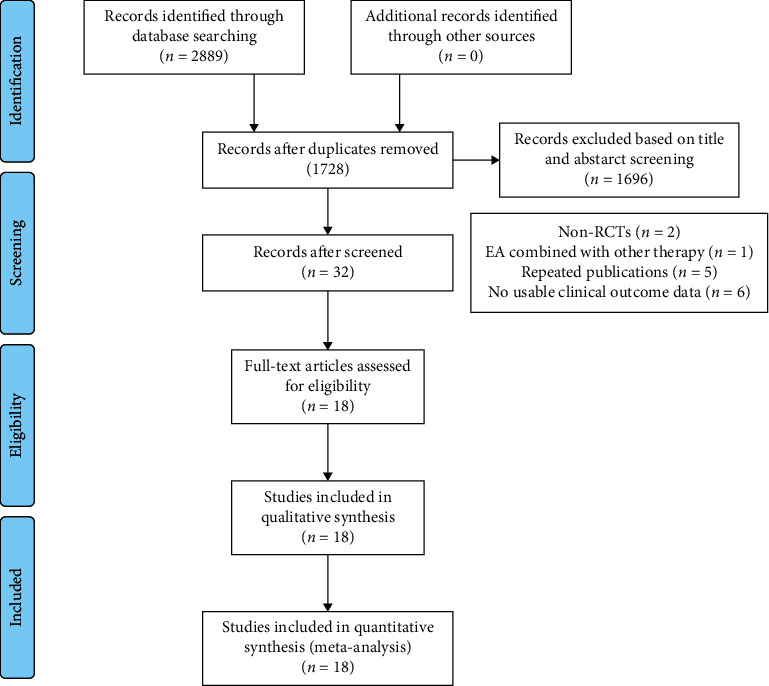
Flowchart of study selection.

A total of 2889 potentially relevant strings were retrieved from the Chinese and English databases. After removing duplicates and screening titles and reading summary, 2857 trials were eliminated. Consequently, the remaining 32 studies were screened by reading full text. Finally, eighteen RCTs [21–38] fulfilled the inclusion criteria, and 1117 subjects including 557 subjects in the electroacupuncture group (EG) and 560 subjects in the control group (CG) were involved. Flowchart of the structured review is illustrated in Figure 1.

### 3.2. Study Characteristics

The included studies involved 1117 subjects who received gynecological surgery. Seven studies [22, 23, 25–27, 32, 33] reported the effects of EA on postoperative gastrointestinal function recovery for patients undergoing total abdominal hysterectomy, and eleven studies [21, 24, 28–31, 34–38] involved laparoscopic surgery. Anesthesia type included general anesthesia and epidural anesthesia [25, 33]. In addition, EA was performed at 24 h prior to surgery in three studies [16, 21, 30], at 30 min prior to surgery or before the start of surgery in ten studies [22, 24, 28, 29, 31, 34–38], and after surgery in five studies [23, 25, 27, 32, 33]. Among all the acupoints involved in the included studies, Zusanli (ST36) (14/18) and Neiguan (PC6) (11/15) were most frequently selected, while less frequently selected acupoints included Hegu (LI4) (4/18), Shangjuxu (ST37) (3/18), Zhongwan (RN12) (3/18), Tianshu (ST25) (3/18), Liangqiu (ST34) (2/18), Sanjinjiao (SP6) (2/18), Liangmen (ST21) (2/18), Xuehai (SP10) (1/18), and Taichong (LR3) (1/18). Tables 1 and 2 presented the characteristics of each included study.

### 3.3. Risk of Bias in Included Studies

**Figure 2:**
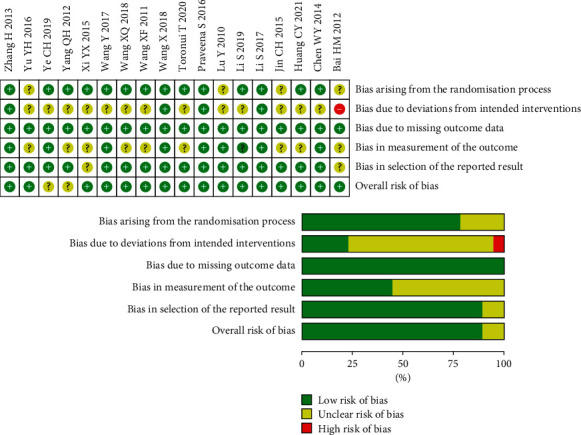
Risk of bias graph.

The detailed results are depicted in Figure 2. All the included studies were reported as random generation, and four of the RCTs [21, 22, 29, 38] were conducted with concealed allocation. Participants were blinded to the group allocation in four studies [21, 22, 29, 38], and assessment blinding was reported in seven RCTs [21, 22, 24, 27, 29, 36, 38]. Meanwhile, based on the evaluation of PEDro, all the scores of the included studies ranged from 5 to 9 (mean score + standard deviation = 6.61 ± 1.42; Supplementary Table 1), which indicated that the methodological quality of included studies was fair to high. All the included studies were described as random generation, while blinding was recorded only in four trials [21, 22, 29, 38]. Additionally, none of the studies reported a follow-up rate of more than 15%, and intention-to-treat analysis was not found in any of the included studies.

### 3.4. Meta-Analysis

#### 3.4.1. Primary Outcomes

**Figure 3:**
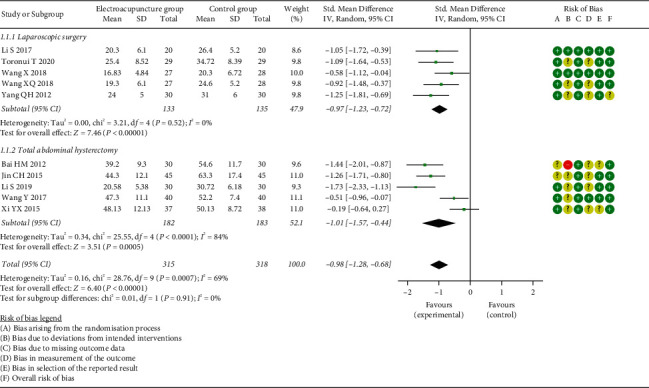
Meta-analysis and forest plot for time to first flatus.

**Figure 4:**
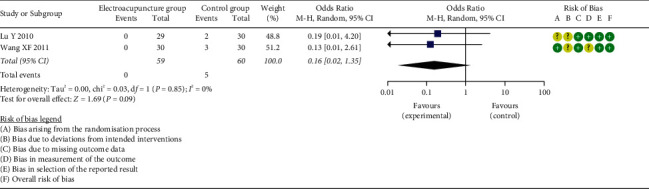
Meta-analysis and forest plot for ratio of time to first flatus >72 h.

*(1) Time to First Flatus*. Ten of the included studies reported TFF [21, 23, 25, 26, 28–30, 32–34], and result from meta-analysis suggested that, overall, EG showed a significantly effect on TFF compared to the CG (SMD = −0.98, 95% CI: [−1.28, −0.68], *P* < 0.00001, *I*^2^ = 69%). Moreover, TFF was significantly shorter in the EG than that in the CG, either for laparoscopic surgery (SMD = −0.97, 95% CI: [−1.23, −0.72], *P* < 0.00001, *I*^2^ = 0%) or total abdominal hysterectomy (SMD = −1.01, 95% CI: [−1.57, −0.44], *P*=0.0005, *I*^2^ = 84%) (Figure 3). Furthermore, we conducted the subgroup analysis by anesthesia types, intervention points, and comparators. All the results supported that EA shortened TFF, no matter for general anesthesia (SMD = −1.01, 95% CI: [−1.37, −0.65], *P* < 0.00001, *I*^2^ = 70%) or epidural anesthesia (SMD = −0.88, 95% CI: [−1.61, −0.16], *P*=0.02, *I*^2^ = 81%) (Supplementary Figure 2), EA applied at 24 h prior to surgery (SMD = −1.23, 95% CI: [−1.73, −0.73], *P* < 0.00001, *I*^2^ = 51%), 30 min prior to surgery (SMD = −0.97, 95% CI: [−1.37, −0.57], *P* < 0.00001, *I*^2^ = 36%), or after surgery (SMD = −0.84, 95% CI: [−1.41, −0.26], *P*=0.004, *I*^2^ = 83%) (Supplementary Figure 3). And a shorter TFF was observed in the EG than that in the CG by comparators as EA VS control (SMD = −1.27, 95% CI: [−1.52, −1.03], *P* < 0.00001, *I*^2^ = 0%), EA + other therapy vs. other therapy (SMD = −1.40, 95% CI: [−2.03, −0.77], *P* < 0.0001, *I*^2^ = 84%), and EA vs. ginger partitioned moxibustion on umbilicus (SMD = −0.35, 95% CI: [−0.67, −0.03], *P*=0.03, *I*^2^ = 1%) (shown in Supplementary Figure 4). However, there was no significant difference in the ratio of subjects with TFF >72 h (OR = 0.16, 95% CI: [0.02, 1.35], *P*=0.09, *I*^2^ = 0%) (Figure 4).

**Figure 5:**
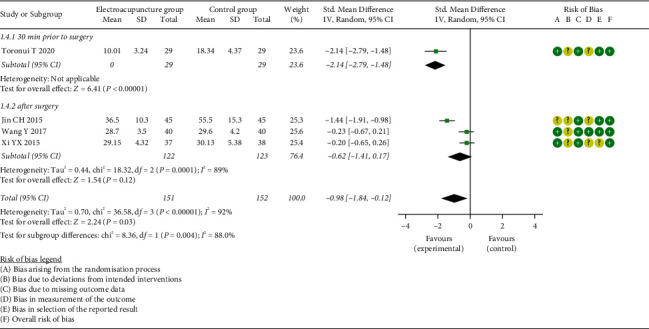
Meta-analysis and forest plot for time to bowel sounds recovery.

*(2) Time to Bowel Sounds Recovery*. Meta-analysis of four studies showed a significantly shorter TBS for the EG compared to the CG (SMD = −0.98, 95% CI: [−1.84, −0.12], *P*=0.03, *I*^2^ = 92%). Nevertheless, subgroup analysis showed that EG presented a shorter TBS when EA performed at 30 min prior to surgery (SMD = −2.14, 95% CI: [−2.79, −1.48], *P* < 0.00001, *I*^2^ = not applicable), whereas subjects receiving EA therapy after surgery in the EG showed no difference to the CG with respect to TBS (SMD = −0.62, 95% CI: [−1.41, 0.17], *P*=0.12, *I*^2^ = 89%) (Figure 5). In addition, there was no significant difference in TBS between patients receiving EA and ginger partitioned moxibustion on umbilicus (SMD = −0.22, 95% CI: [−0.53, −0.10], *P*=0.18, *I*^2^ = 0%) (Supplementary Figure 5).

**Figure 6:**
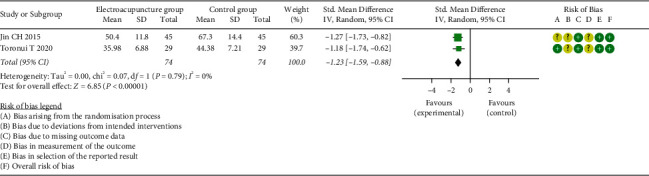
Meta-analysis and forest plot for time to first defecation.

*(3) Time to First Defecation*. In this study, two trials recorded TFD. Meta-analysis result revealed that, compared to the CG, TFD was significantly shorter in the EG (SMD = −1.23, 95% CI: [−1.59, −0.88], *P* < 0.0001, *I*^2^ = 0%) (Figure 6).

#### 3.4.2. Secondary Outcomes

**Figure 7:**
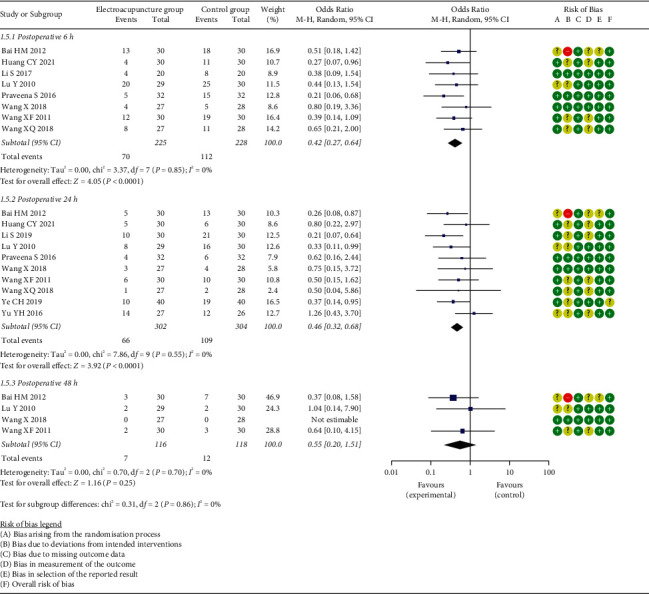
Meta-analysis and forest plot for ratio of PONV.

*(1) Postoperative Nausea and Vomiting (PONV)*. The incidence of PONV at postoperative 6 h, 24 h, and 48 h was reported in eight [21–23, 27, 29–31, 35], ten [21–23, 27, 29–31, 35–37], and four studies [23, 27, 29, 31], respectively. Meta-analysis results suggested a lower incidence of PONV at postoperative 6 h (OR = 0.42, 95% CI: [0.27, 0.64], *P* < 0.0001, *I*^2^ = 0%), 24 h (OR = 0.46, 95% CI: [0.32, 0.68], *P* < 0.0001, *I*^2^ = 0%) in the EG than that in the CG, whereas no significant difference in ratio of PONV at postoperative 48 h (OR = 0.55, 95% CI: [0.20, 1.51], *P*=0.25, *I*^2^ = 0%) was detected between the two groups (Figure 7). Subgroup analysis revealed that no difference in the ratio of PONV at postoperative 6 h was observed between the two groups when EA performed at 24 h prior to surgery (OR = 0.53, 95% CI: [0.22, 1.27], *P*=0.15, *I*^2^ = 0%) or after surgery (OR = 0.48, 95% CI: [0.22, 1.06], *P*=0.07, *I*^2^ = 0%), but its ratio was lower in the EG when EA was performed at 30 min prior to surgery in comparison to the CG (OR = 0.34, 95% CI: [0.19, 0.63], *P*=0.0006, *I*^2^ = 0%). Regarding the ratio of PONV at postoperative 24 h, it was lower in the EG than that in the CG whenever EA was conducted at 24 h (OR = 0.25, 95% CI: [0.09, 0.67], *P*=0.006, *I*^2^ = 0%) or 30 min prior to surgery (OR = 0.46, 95% CI: [0.28, 0.76], *P*=0.03, *I*^2^ = 0%), or after surgery (OR = 0.03, 95% CI: [0.13, 0.67], *P*=0.003, *I*^2^ = 0%). However, there was no significant difference in the ratio of PONV at postoperative 48 h between the EG and CG regardless of the preoperative (OR = 0.64, 95% CI: [0.10, 4.15], *P*=0.64, *I*^2^ = not applicable) or postoperative electroacupuncture treatment (OR = 0.52, 95% CI: [0.16, 1.71], *P*=0.28, *I*^2^ = 0%).

**Figure 8:**
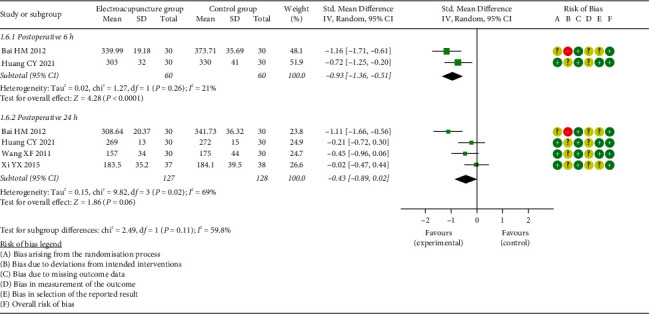
Meta-analysis and forest plot for motilin.

*(2) Motilin (MTL)*. Two studies [23, 35] investigated the effects of EA on MTL at postoperative 6 h, and four studies [23, 31, 33, 35] evaluated the effect at postoperative 24 h. Meta-analysis results suggested that a significant effect in favor of EA on level of MTL at postoperative 6 h (SMD = −0.93, 95% CI: [−1.36, −0.61], *P* < 0.0001, *I*^2^ = 21%), while no significant effect was observed at postoperative 24 h (SMD = −0.43, 95% CI: [−0.89, 0.02], *P*=0.06, *I*^2^ = 69%) in the EG when compared to the CG (Figure 8).

**Figure 9:**
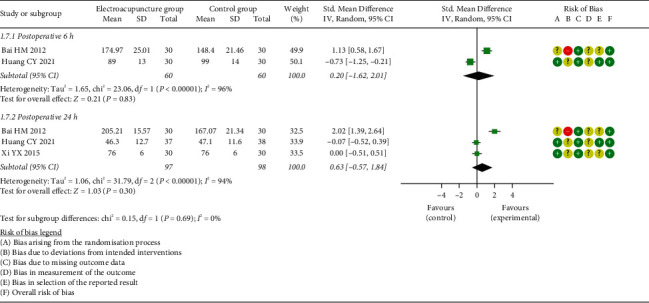
Meta-analysis and forest plot for gastrin.

*(3) Gastrin (GAS)*. Two studies [23, 35] compared patients receiving EA to those in the control condition in terms of GAS at postoperative 6 h, and three studies [23, 33, 35] estimated the effect of EA on GAS at postoperative 24 h. Meta-analysis results revealed that no significant effect on GAS was observed at neither postoperative 6 h (SMD = 0.20, 95% CI: [−1.62, 2.01], *P*=0.83, *I*^2^ = 96%) nor 24 h (SMD = 0.63, 95% CI: [−0.57, 1.84], *P*=0.30, *I*^2^ = 94%) (Figure 9).

**Figure 10:**
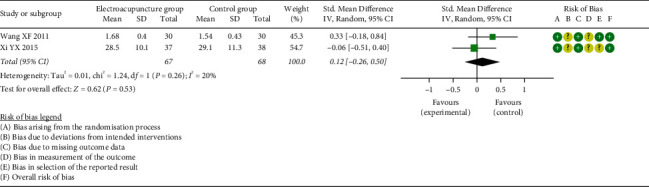
Meta-analysis and forest plot for VIP.

*(4) Vasoactive Intestinal Peptide (VIP)*. Meta-analysis of two studies [31, 33] demonstrated a small, but nonsignificant, overall effect concerning VIP (SMD = 0.12, 95% CI: [−0.26, 0.50], *P*=0.53, *I*^2^ = 20%) (Figure 10).

**Figure 11:**
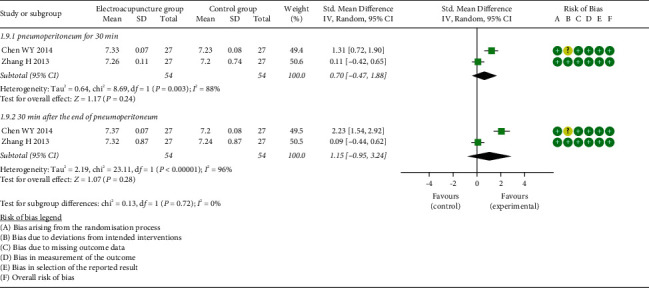
Meta-analysis and forest plot for pHi.

*(5) pH Value of Gastric Mucosa (pHi)*. Meta-analysis of two studies [24, 38] indicated that no significant difference in pHi was determined between the two groups neither at pneumoperitoneum for 30 min (SMD = 0.70, 95% CI: [−0.47, 1.88], *P*=0.24, *I*^2^ = 88%) nor at 30 min after the end of pneumoperitoneum (SMD = 1.15, 95% CI: [−0.95, 3.24], *P*=0.28, *I*^2^ = 96%) (Figure 11).

**Figure 12:**
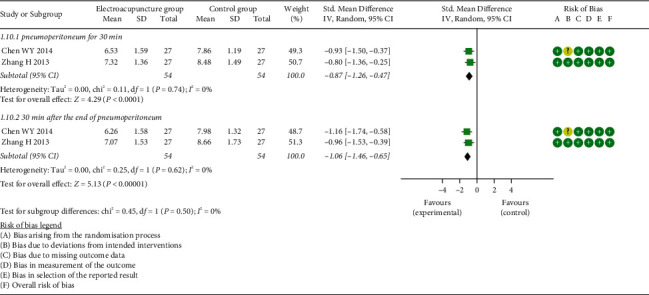
Meta-analysis and forest plot for PgCO_2_.

*(6) Gastric Mucosal Partial Pressure of Carbon Dioxide (PgCO_2_)*. Two studies examined the effect of EA on PgCO_2_. Meta-analysis results revealed that a large significant effect on decreasing PgCO_2_ was found in the EG VS CG, both at pneumoperitoneum for 30 min (SMD = −0.87, 95% CI: [−1.26, −0.47], *P* < 0.0001, *I*^2^ = 0%) and 30 min after the end of pneumoperitoneum (SMD = −1.06, 95% CI: [−1.46, −0.65], *P* < 0.00001, *I*^2^ = 0%) (Figure 12).

*(7) Hospital Stay*. In the study, hospital stay was recorded in only one study [30], which reported that electroacupuncture applied at 24 h prior to surgery showed no significant effect on the duration of hospital stay (EG vs. CG: (3.6 ± 0.4) *d* vs. (3.9 ± 0.2) *d*, *P*=0.492).

*(8) Adverse Event*. One participant in one study [21] reported pain at the needling sites and bruising, while the side effects were alleviated spontaneously without any medical assistance. In addition, it was reported in two studies [30, 38] that three participants withdrew because they were not intolerant to EA.

### 3.5. Sensitivity Analysis

In this study, we conducted the sensitivity analysis to evaluate the robustness of primary outcome indicators with high heterogeneity by removing studies from the analysis individually. After sensitivity analysis for TFF, no substantial change about overall heterogeneities and results was detected (Supplementary Table 2). Meanwhile, we performed sensitivity analysis for TBS, and the results were listed in Supplementary Table 3. The overall effect indicated no statistically significant difference when the study reported by Jin and Jing [25] (SMD = −0.83, 95% CI: [−1.09, 0.24], *P*=0.13, *I*^2^ = 93%) or Toronui [28] (SMD = −0.62, 95% CI: [−1.41, 0.17], *P*=0.12, *I*^2^ = 89%) was eliminated. However, the results of subgroup analysis by intervention time points for the remaining studies [28, 32, 33] showed no substantial change. It was found that TBS was significantly shorter in the EG than that in the CG when EA was received prior to surgery than that in the CG (SMD = −2.14, 95% CI: [−2.79, −1.48], *P* < 0.00001, *I*^2^ = not applicable), whereas no significant difference was detected with no heterogeneity when EA was applied after surgery (SMD = −0.22, 95% CI: [−0.53, 0.10], *P*=0.18, *I*^2^ = 0%).

### 3.6. Publication Bias

**Figure 13:**
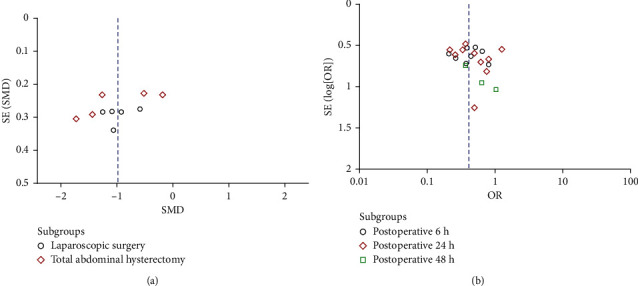
Publication bias assessed by funnel plots for TFF (a) and PONV (b).

In the present study, Begg's test and Egger's test were employed to examine publication bias for the indices recorded more than 2 included trails. It was suggested that there should be no publication bias except for MTL at postoperative 24 h (*P*=0.042) (Supplementary Table 4). Moreover, visual inspection of funnel plots seemed to be relatively symmetrical for TFF and PONV (Figure 13), indicating no evidence for publication bias.

### 3.7. Evidence Quality Assessment According to GRADE

Based on GRADE guidelines, we evaluated the quality of evidence from five aspects, including risk of bias, inconsistency, indirectness, imprecision, and publication bias. During the treatment period, EA therapists directly contacted to participants, which led to the high risk of performance bias, and consequently lowered the evidence quality. In this study, there was low evidence in TFF, TFD and PONV, and very low evidence in the remaining. Details were recorded in Supplementary Table 5.

## 4. Discussion

In spite of continuous improvement for surgical approach and technique, postoperative gastrointestinal dysfunction still commonly occurred after gynecological surgery. To resolve this clinical concern, EA has been proposed to promote gastrointestinal function recovery, while relevant evidence is sparse. To the best of our knowledge, this is first systematical review and meta-analysis regarding the efficacy and safety of EA on recovery of gastrointestinal function for patients receiving gynecological surgery.

We found that EA was able to shorten the time to first flatus, first defecation after gynecological surgery. These findings were in accordance with those reported by the previous meta-analyses [11–14] for other patient populations, but there were some new findings after subgroup analysis in the current study. Our findings suggested that EA was beneficial to reduce the time to first of bowel sounds recovery when EA was used at 30 min prior to surgery. However, no significant effect on TBS was observed when patients undergoing gynecological surgery received EA treatment after surgery. Additionally, the evidence in favor of EA for decreasing the ratio of PONV was observed only at postoperative 6 h and 24 h, which echo the results reported by Lee et al. [39], while the benefits were unobvious at postoperative 48 h. Similarly, the effect of EA on MTL was detected only within postoperative 6 h. Thus, our findings showed that the benefits of EA for PONV and MTL concentrate within 24 h after surgery, and even earlier. MTL stimulated gastrointestinal motility and accelerated gastric emptying, which could induce or aggravate nausea and vomiting [40, 41]. Thus, it could be the reason that EA decreased MTL level and consequently resulted in lowering the ratio of PONV at with postoperative 24 h. After subgroup analysis for PONV by intervention points, we found that EA presented a largest effect in lowering the ratio of PONV when it was applied at 30 min prior to surgery, whereas no or very small effect was found when patients were treated with EA after gynecological surgery. It might be the reason that factors contributing to postoperative gastrointestinal dysfunction mainly generated during intraoperative period; it should be more proper to applied EA before the start of surgery until the end of surgery, while the effectiveness of EA would be weakened when used too early, and postoperative application seems to be late.

PgCO_2_ is a sensitive indicator reflecting the changes of blood oxygen in gastric mucosa [42]. It was proved that pHi was closely related with intestinal oxygen consumption, and decreased gastric pHi level prognosticated morbidity and mortality [43]. The results of this meta-analysis revealed that EA deceased the PgCO_2_ both at 30 min after the start of pneumoperitoneum and 30 min after the end of pneumoperitoneum, whereas it was ineffective for mediation of pHi after surgery. Additionally, GAS was the hormone primarily responsible for gastric acid secretion [44], and VIP was a gut peptide hormone regulating gut motility [45]; hence, they were associated with postoperative gastrointestinal function [46]. However, EA showed no significant effect on GAS and VIP after gynecological surgery. Therefore, EA accelerated the recovery of postoperative gastrointestinal function through other pathways, not depending on the mediation of GAS or VIP.

Several potential limitations should be considered in the present review had. Firstly, the EA parameters including acupoints, and times and frequency of electroacupuncture were selected without a consolidated standard in the included studies, which could be a potential source of clinical heterogeneity; secondly, blind methods of the included studies were rarely provided in detail; thirdly, as was reported in the previous basic experimental studies, EA mediated of the autonomic nervous system, improved dysmotility and local inflammation, and consequently ameliorated POI to restore gastrointestinal function [47], and the therapeutic effects was different when using lower limb and abdomen acupoints [48]. However, few clinical trials focus on examining the different effect of EA by using different acupoints.

Given the limitations of this work, large-scale RCTs with more rigorous and robust methods are still needed in future studies. Most importantly, the majority of the studies were not blind to participants and acupuncturists; sham-controlled studies should be performed to avoid performance bias. Hence, more studies should be conducted strictly following standard reporting guidelines such as CONSORT [49]. Furthermore, more RCTs in the future should investigate the therapeutic effects of EA using different acupoints in order to identify the definitely effective acupoints.

## 5. Conclusion

In this analysis, we systematically reviewed and quantified the effect of EA on gastrointestinal function after gynecological surgery. Overall, EA was an effective and safe treatment for promoting recovery of postoperative gastrointestinal function, such as shortening TFF and TFD, TBS, regulating MTL, and decreasing the ratio of PONV within postoperative 24h, for patients receiving gynecological surgery through abdominal and laparoscopic approaches, while the effects on MTL and PONV varied with different intervention points, and EA used at 30 min prior to surgery might be recommended. Moreover, EA could regulate PgCO_2_ during anesthesia process, which was associated with the recovery of postoperative gastrointestinal function. However, EA exerted no significant impact on mediating GAS, VIP, and pHi. Thus, EA could be a promising strategy for the prevention and treatment of gastrointestinal dysfunction after gynecological surgery. Notably, evidence quality ranged from low to very low; large-scale and high-quality RCTs were needed.

## Figures and Tables

**Table 1:** Characteristics of the included studies.

First author (year)	Age (years)		Sample size	Type of surgery	Type of anesthesia	Duration of anesthesia/surgery	
	EG	CG	EG/CG			EG	CG
Li et al. (2017) [21]	35.2 ± 6.1	34.4 ± 9.1	20/20	Laparoscopic surgery	General anesthesia (I/II)	89.3 ± 38.9	95.5 ± 32.8
Praveena et al. (2016) [22]	47.5 ± 7.94	48.72 ± 6.72	32/32	Total abdominal hysterectomy	General anesthesia (I/II)	149.06 ± 42.64	151.97 ± 50.71
Bai et al. (2012) [23]	42∼60		30/30	Total abdominal hysterectomy	General anesthesia (I/II)	—	—
Chen et al. (2014) [24]	18∼65		27/27	Laparoscopic surgery	General anesthesia (I/II)	95.2 ± 39.4	77.8 ± 23.8
Jin and Jing (2015) [25]	35∼66 (48.3 ± 6.3)		45/45	Total abdominal hysterectomy	Epidural anesthesia	―	―
Li et al. (2019) [26]	37.26 ± 8.83	36.96 ± 9.05	30/30	Total abdominal hysterectomy	General anesthesia (I/II)	—	—
Lu et al. (2010) [27]	40∼60		29/30	Total abdominal hysterectomy	General anesthesia (I/II)	―	―
Toronui (2019) [28]	40.54 ± 5.82	40.62 ± 5.84	29/29	Laparoscopic surgery	General anesthesia	84.31 ± 35.21	84.58 ± 35.56
Wang et al. (2018) [29]	35.76 ± 7.15	34.88 ± 7.28	27/28	Laparoscopic surgery	General anesthesia (I/II)	68.69 ± 29.34	67.62 ± 34.42
Wang et al. (2018) [30]	34.9 ± 5.1	34.8 ± 6.2	27/28	Laparoscopic surgery	General anesthesia (I/II)	80.2 ± 36.9	85.3 ± 31.8
Wang et al. (2011) [31]	41.2 ± 6.1	39.3 ± 8.5	30/30	Laparoscopic surgery	General anesthesia (I/II)	67.2 ± 16.0	70.0 ± 10.2
Wang and Xi (2017) [32]	31.3 ± 4.5	31.5 ± 4.3	40/40	Total abdominal hysterectomy	—	138 ± 24	132 ± 30
Xi and Wang (2015) [33]	33 ± 3	33 ± 3	37/38	Total abdominal hysterectomy	Epidural anesthesia	103.2 ± 30.6	102.6 ± 31.8
Yang et al. (2012) [34]	32 ± 9	30 ± 10	30/30	Laparoscopic surgery	General anesthesia (I/II)	78 ± 23	80 ± 25
Huang et al. (2021) [35]	31∼56		30/30	Laparoscopic surgery	General anesthesia (I/II)	―	―
Ye and Huang (2019) [36]	33 ± 9	35 ± 10	40/40	Laparoscopic surgery	General anesthesia (I/II)	97 ± 9	95 ± 10
Yu and Ning (2016) [37]	31 ± 4.4	30 ± 5.4	27/26	Laparoscopic surgery	General anesthesia (I/II)	78.1 ± 16.2	75.0 ± 13.7
Zhang et al. (2013) [38]	34.74 ± 5.64	36.81 ± 9.26	27/27	Laparoscopic surgery	General anesthesia (I/II)	—	—

**Table 2:** Interventions, outcomes, and study design on the included studies.

First author (year)	Intervention	Intervention parameters	Intervention dose	Main outcome	Study design
Li et al. (2017) [21]	EG : EA + routine treatment; CG: routine treatment.	Acupoints: bilateral Neiguan (PC6) and Zusanli (ST36), mode: dense-disperse wave; frequency: 20/100 Hz; intensity (mA) : strong but comfortable. 7548	24 hours prior to the surgery, once for 30 min.	TFF, PONV	RCT
Praveena et al. (2016) [22]	EG : EA + routine treatment, CG: routine treatment.	acupoints: bilateral Hegu (LI4) and Neiguan (PC6), mode: continuous wave, frequency: 2 Hz; intensity (mA): Level 1.	Before the start of surgery until the end of surgery.	PONV	RCT
Bai et al. (2012) [23]	EG : EA + Tropisetron (5 mg, intravenous injection after anesthesia induction) + routine treatment; CG : Tropisetron (5 mg, intravenous injection after anesthesia induction) + routine treatment.	Acupoints: bilateral Neiguan (PC6) and Zusanli (ST36), Shangjuxu (ST37), Zhongwan (RN12), Tianshu (ST25), mode: unreported; frequency: 2 Hz; intensity (mA): unclear.	At hour 5, 23, and 27 after surgery, 30 min/once.	PONV, TFF, MTL, GAS	RCT
Chen et al. (2014) [24]	EG : EA + routine treatment; CG: routine treatment	Acupoints: bilateral Liangqiu (ST34) and Zusanli (ST36), mode: continuous wave; frequency: 2 Hz; intensity (mA): maximum tolerable.	30 minutes prior to the surgery until the end of surgery.	PHi, PgCO_2_	RCT
Jin and Jing (2015) [25]	EG : EA + routine treatment; CG: routine treatment	Acupoints: bilateral Zusanli (ST36); mode: unreported; frequency and intensity (mA): unclear.	At hour 5 after surgery, twice a day, until time to first flatus.	TBS, TFF, TFD	RCT
Li et al. (2019) [26]	EG : EA + routine treatment; CG: routine treatment.	Acupoints: bilateral Neiguan (PC6), Xuehai (SP10), Hegu (LI4) and Zusanli (ST36), mode: dense-disperse wave; frequency: 2/10/50/100 Hz; intensity (mA): unclear.	24 hours prior to the surgery, once for 30 min.	TFF, PONV,	RCT
Lu et al. (2010) [27]	EG : EA + Tropisetron (5 mg, intravenous injection prior to the end of surgery) + routine treatment; CG : Tropisetron (5 mg, intravenous injection prior to the end of surgery) + routine treatment.	Acupoints: bilateral Neiguan (PC6) and Zusanli (ST36), Hegu (LI4), Sanyinjiao (SP6), Taichong (LR3), mode: dense-disperse wave; frequency: 2/10 Hz; intensity (mA): maximum tolerable.	At hours 1, 5, and 23 after surgery, 30 min/once.	PONV, number of TFF more than 72 h	RCT
Toronui (2020) [28]	EG : EA + routine treatment; CG: routine treatment.	Acupoints: bilateral Zusanli (ST36), frequency: 2/10 Hz.	Prior to the surgery, once for 15 min.	TBS, TFF, TFD, PONV	RCT
Wang et al. (2018) [29]	EG : EA + Tropisetron (5 mg, intravenous injection prior to the end of surgery) +routine treatment; CG : Tropisetron (5 mg, intravenous injection prior to the end of surgery) + routine treatment.	Acupoints: bilateral Neiguan (PC6) and Zusanli (ST36), mode: dense-disperse wave; frequency: 2 Hz; intensity (mA): maximum tolerable.	30 minutes prior to the surgery, once for 30 min.	PONV, TFF	RCT
Wang et al. (2018) [30]	EG : EA + Tropisetron (5 mg, intravenous injection prior to the end of surgery) + routine treatment; CG : Tropisetron (6 mg, intravenous injection prior to the end of surgery) + routine treatment.	Acupoints: bilateral Neiguan (PC6) and Zusanli (ST36), mode: dense-disperse wave; frequency: 2 Hz; intensity (mA): maximum tolerable.	24 hours prior to the surgery, once for 30 min.	TFF, PONV	RCT
Wang et al. (2011) [31]	EG : EA + routine treatment; CG: routine treatment.	Acupoints: bilateral Zusanli (ST36), Neiguan (PC6), mode: continuous wave; frequency: 2 Hz; intensity (mA): maximum tolerable.	Prior to the surgery, once for 15 min.	PONV, MTL, VIP, number of TFF more than 72 h	RCT
Wang and Xi (2017) [32]	EG : EA + routine treatment; CG : Ginger partitioned moxibustion on umbilicus + routine treatment.	Acupoints: bilateral Liangmen (ST21) and Zusanli (ST36), Shangjuxu (ST37), Zhongwan (RN12), Tianshu (ST25), mode: continuous wave.	After surgery, once for 30 min, once a day, for 3 d.	TBS, TFF	RCT
Xi and Wang (2015) [33]	EG : EA + routine treatment; CG1 : Ginger partitioned moxibustion on umbilicus + routine treatment	Acupoints: bilateral Liangmen (ST21) and Zusanli (ST36), Shangjuxu (ST37), Zhongwan (RN12), Tianshu (ST25), mode: continuous wave; intensity (mA): maximum tolerable.	After surgery, once for 30 min, once a day, for 3 d.	TBS, TFF, MTL, GAS,	
VIP	RCT				
Yang et al. (2012) [34]	EG : EA + routine treatment; CG: routine treatment.	Acupoints: bilateral Sanyinjiao (SP6) and Zusanli (ST36), mode: dense-disperse wave; frequency: 2/100 Hz; intensity (mA): maximum tolerable.	30 minutes prior to the surgery until the end of surgery.	TFF	RCT
Huang et al. (2021) [35]	EG : EA + routine treatment; CG: routine treatment.	Acupoints: bilateral Neiguan (PC6), mode: dense-disperse wave; frequency: 3/20 Hz; intensity (mA): unclear.	Prior to the surgery, once for 20 min.	PONV, MTL, GAS	RCT
Ye and Huang (2019) [36]	EG : EA + routine treatment; CG: routine treatment.	Acupoints: bilateral Neiguan (PC6), mode: unreported, frequency: 3/21 Hz; intensity (mA): comfortable.	Prior to the surgery, once for 20 min.	PONV	RCT
Yu and Ning (2016) [37]	EG : EA + Tropisetron (2 mg, intravenous injection prior to the end of surgery) + routine treatment; CG1 : Tropisetron (2 mg, intravenous injection prior to the end of surgery) + routine treatment.	Acupoints: bilateral Neiguan (PC6), and Hegu (LI4), mode: dense-disperse wave; frequency: 20/100 Hz; intensity (mA): 10 mA.	Prior to the surgery, once for 30 min.	PONV	RCT
Zhang et al. (2013) [38]	EG : EA + routine treatment; CG: routine treatment.	Acupoints: bilateral Liangqiu (ST34) and Zusanli (ST36), mode: continuous wave; frequency: 2 Hz; intensity (mA): maximum tolerable.	30 minutes prior to the surgery until the end of surgery.	pHi, PgCO_2_	RCT

EG: electroacupuncture group; CG: control group; EA: electroacupuncture; TFF: time to first flatus; TFD: time first to defecation; TFBS: time to first bowel sound; PONV: postoperative nausea and vomiting; MTL: motilin; GAS: gastrin; pHi : PH value of gastric mucosa; PgCO_2_: gastric mucosal partial pressure of carbon dioxide; VIP: vasoactive intestinal peptide; and RCT: randomized controlled trials.

## Data Availability

The original data analyzed in this study are presented in the article; further inquiries should be directed to the corresponding authors.

## Supplementary Materials

Supplementary Materials
*Supplementary file 1: Supplementary Figure 1*. The search strategy in this review. *Supplementary Figure 2*. Forest plot of time to first flatus by subgroup analysis for anesthesia type. *Supplementary Figure 3*. Forest plot of time to first flatus by subgroup analysis for intervention points. *Supplementary Figure 4*. Forest plot of time to first flatus by subgroup analysis for comparators. *Supplementary Figure 5*. Forest plot of time to bowel sounds recovery by subgroup analysis for comparators. *Supplementary Table 1*. Sensitivity analysis for time to first flatus. *Supplementary Table 1*. PEDro scores of the included studies. *Supplementary Table 2*. Sensitivity analysis for time to first flatus. *Supplementary Table 3*. Evidence quality assessment according to GRADE. *Supplementary file 2*. PRISMA checklist.
