# Supplementary material for: Electroacupuncture for Gastrointestinal Function Recovery after Gynecological Surgery: A Systematic Review and Meta-Analysis
Source: Evid Based Complement Alternat Med. 2021 Dec 21;2021:8329366. doi: 10.1155/2021/8329366 (PMC8714373; doi:10.1155/2021/8329366)
Supplement: Supplementary Materials — Supplementary file 1: Supplementary Figure 1. The search strategy in this review. Supplementary Figure 2. Forest plot of time to first flatus by subgroup analysis for anesthesia type. Supplementary Figure 3. Forest plot of time to first flatus by subgroup analysis for intervention points. Supplementary Figure 4. Forest plot of time to first flatus by subgroup analysis for comparators. Supplementary Figure 5. Forest plot of time to bowel sounds recovery by subgroup analysis for comparators. Supplementary Table 1. Sensitivity analysis for time to first flatus. Supplementary Table 1. PEDro scores of the included studies. Supplementary Table 2. Sensitivity analysis for time to first flatus. Supplementary Table 3. Evidence quality assessment according to GRADE. Supplementary file 2. PRISMA checklist. [file 8329366.f1.zip › 8329366.f1/revised 2-supplementry file 1 (1).pdf]

Pubmed

((randomized controlled trial[Publication Type] OR randomized[Title/Abstract] OR placebo[Title/Abstract]) AND (((((((((((PONV) OR (Nausea and Vomiting, Postoperative)) OR Vomiting, Postoperative) OR Postoperative Emesis) OR Postoperative Vomiting) OR Emesis, Postoperative) OR Emeses, Postoperative) OR Postoperative Emeses) OR Postoperative Nausea) OR Nausea, Postoperative)) OR (((((((POI) OR (postoperative gastrointestinal motility disorder))) OR (postoperative gastrointestinal function recovery)) OR (postoperative gastrointestinal dysfunction)) OR (postoperative ileus)) OR (postoperative gastrointestinal function)))) AND ((electroacupuncture) OR (acupuncture)) 286

Embase

No.	Query	Results
#12	#9 AND #10 AND #11	501
#11	'randomized controlled trial'/exp OR 'controlled clinical trial'/exp OR 'randomized':ti,ab OR 'placebo':ti,ab OR 'drug therapy':lnk OR 'randomly':ti,ab OR 'trial':ti,ab OR 'groups':ti,ab	7897247
#10	#5 OR #6 OR #7 OR #8	57569
#9	#1 OR #2 OR #3 OR #4	21517
#8	'acupuncture'	54994
#7	'acupuncture'/exp	49652
#6	'electroacupuncture'	8248
#5	'electroacupuncture'/exp	7345
#4	'postoperative nausea and vomiting' OR 'ponv' OR 'nausea and vomiting, postoperative' OR 'vomiting, postoperative' OR 'postoperative emesis' OR 'postoperative vomiting' OR 'emesis, postoperative' OR 'emeses, postoperative' OR 'postoperative emeses' OR 'postoperative nausea' OR 'nausea, postoperative'	13229
#3	'postoperative nausea and vomiting'/exp	10956
#2	'poi' OR 'postoperative gastrointestinal function recovery' OR 'postoperative gastrointestinal dysfunction' OR 'postoperative ileus' OR 'postoperative gastrointestinal function'	8535
#1	'postoperative ileus'/exp	3346

Cochran

ID	Search	Hits

#1	((((((((((PONV) OR (Nausea and Vomiting, Postoperative)) OR Vomiting, Postoperative) OR Postoperative Emesis) OR Postoperative Vomiting) OR Emesis, Postoperative) OR Emeses, Postoperative) OR Postoperative Emeses) OR Postoperative Nausea) OR Nausea, Postoperative)):ti,ab,kw (Word variations have been searched)	14927
#2	(((((((POI) OR (postoperative gastrointestinal motility disorder))) OR (postoperative gastrointestinal function recovery)) OR (postoperative gastrointestinal dysfunction)) OR (postoperative ileus)) OR (postoperative gastrointestinal function)):ti,ab,kw (Word variations have been searched)	2761
#3	("electroacupuncture"):ti,ab,kw (Word variations have been searched)	2665
#4	(acupuncture):ti,ab,kw (Word variations have been searched)	15475
#5	#1 OR #2	17042
#6	#3 OR #4	16261
#7	#5 AND #6	480

CNKI database:

(Su ='electroacupuncture 'and (Su ='intestinal obstruction' or Su ='gastrointestinal function 'or Su ='vomiting' or Su ='nausea ')) or (TKA ='electroacupuncture' and (TKA ='intestinal obstruction 'or TKA ='gastrointestinal function' or TKA ='vomiting 'or TKA ='nausea') (exact match) 1010

Wangfang database:

(title or key words: (electroacupuncture) or title or key words: (acupuncture)) and title or key words: (postoperative) and (title or key words: (intestinal obstruction) or title or key words: (gastrointestinal function) or title or key words: (nausea) or title or key words: (vomiting)) 549

VIP database (www.cqvip.com):

M = (electroacupuncture or acupuncture) and M = postoperative and M = (intestinal obstruction or gastrointestinal function or nausea or vomiting) 63

Supplementary eFigure 1 The search strategy in this review.

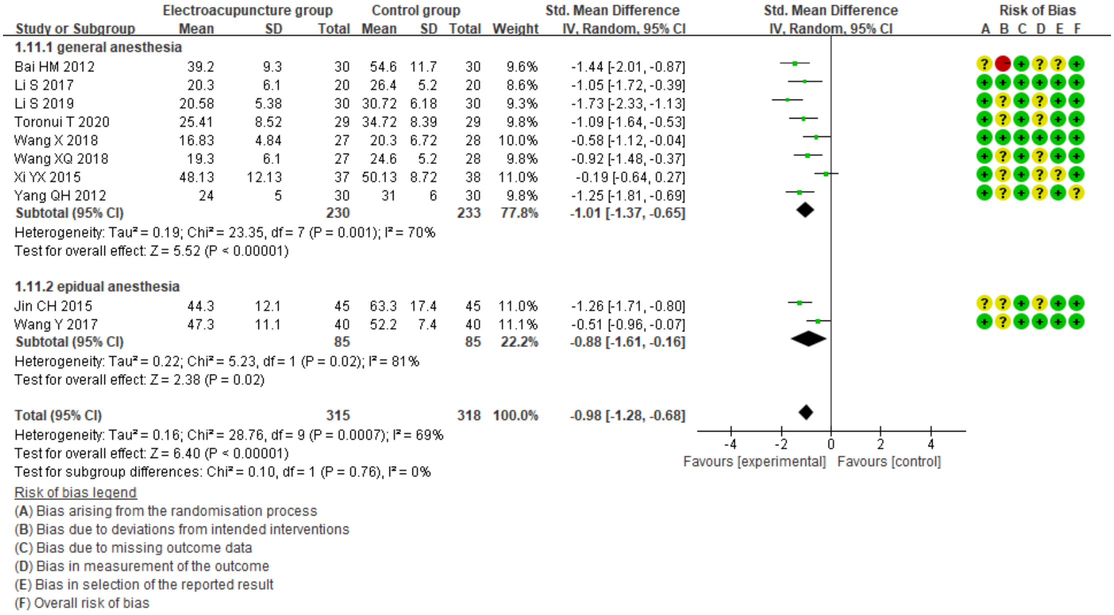

Supplementary eFigure 2 Forest plot of time to first flatus by subgroup analysis for anesthesia type.

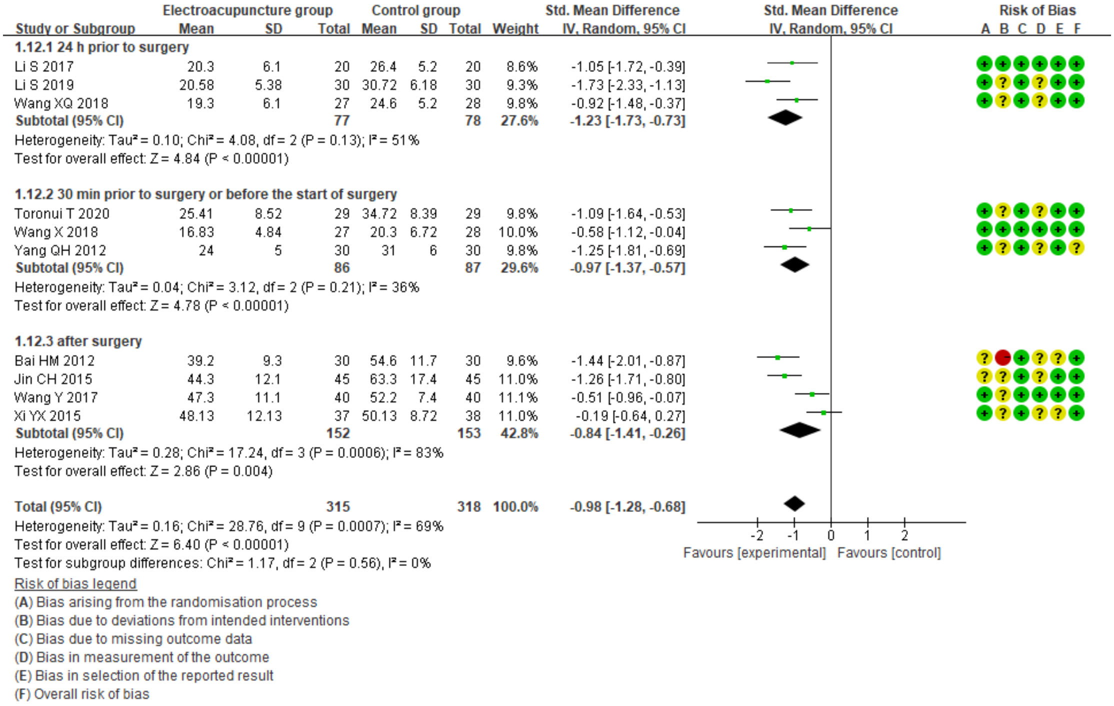

Supplementary eFigure 3 Forest plot of time to first flatus by subgroup analysis for intervention points.

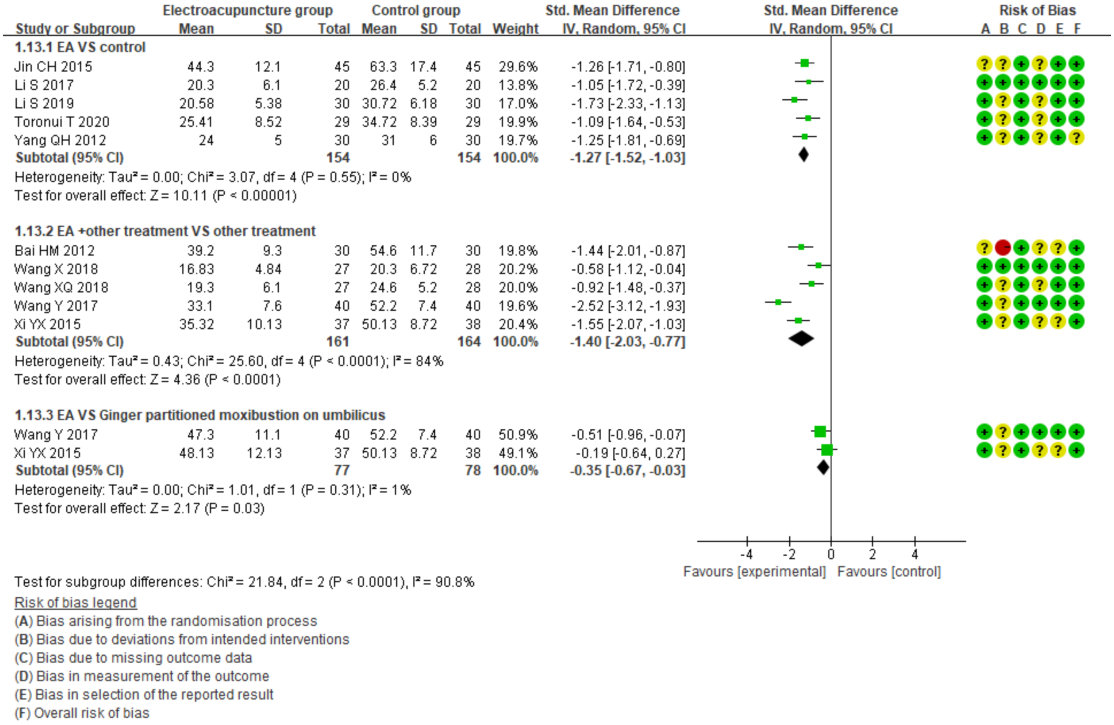

Supplementary eFigure 4 Forest plot of time to first flatus by subgroup analysis for comparators.

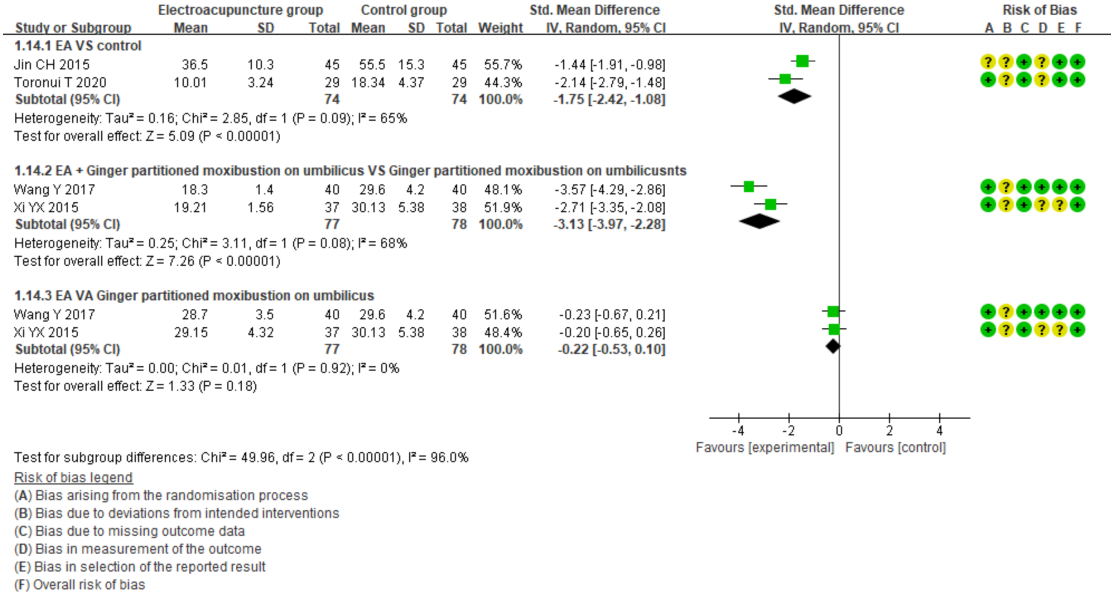

Supplementary eFigure 5 Forest plot of time to bowel sounds recovery by subgroup analysis for comparators.

Supplementary eTable 1 PEDro scores of the included studies.

Study	Random Allocation	Concealed Allocation	Similar Baseline	Blinding Subjects	Blinding Therapists	Blinding Assessors	Dropout <15%	Intention to Treat	Between-group Statistics	Point Measures	Total Score
Li S 2017[21]	Yes	Yes	Yes	Yes	No	Yes	Yes	Yes	Yes	Yes	9
Praveena 2016[22]	Yes	Yes	Yes	Yes	No	Yes	Yes	Yes	Yes	Yes	9
Bai HM 2012[23]	Yes	No	Yes	No	No	No	Yes	Yes	Yes	Yes	6
Chen WY 2014[24]	Yes	No	Yes	No	No	Yes	Yes	Yes	Yes	Yes	7
Jin CH 2015[25]	Yes	No	Yes	No	No	No	Yes	Yes	Yes	Yes	6
Li S 2019[26]	Yes	No	Yes	No	No	No	Yes	Yes	Yes	Yes	6
Lu Y 2010[27]	Yes	No	Yes	No	No	Yes	Yes	No	Yes	Yes	6
Toronui T 2020[28]	Yes	No	Yes	No	No	No	Yes	Yes	Yes	Yes	6
Wang X 2018[29]	Yes	Yes	Yes	Yes	No	Yes	Yes	Yes	Yes	Yes	9
Wang XQ 2018[30]	Yes	No	Yes	No	No	No	Yes	Yes	Yes	Yes	6
Wang XF 2011[31]	Yes	No	Yes	No	No	No	Yes	No	Yes	Yes	5
Wang Y 2017[32]	Yes	No	Yes	No	No	No	Yes	Yes	Yes	Yes	6
Xi YX 2015[33]	Yes	No	Yes	No	No	No	Yes	No	Yes	Yes	5
Yang QH 2012[34]	Yes	No	Yes	No	No	No	Yes	No	Yes	Yes	5
Huang CY 2021[35]	Yes	No	Yes	No	No	No	Yes	Yes	Yes	Yes	6
Ye CH 2019[36]	Yes	No	Yes	No	No	Yes	Yes	Yes	Yes	Yes	7
Yu YH 2016[37]	Yes	No	Yes	No	No	No	Yes	Yes	Yes	Yes	6
Zhang H 2013[38]	Yes	Yes	Yes	Yes	No	Yes	Yes	Yes	Yes	Yes	9

PEDro: Physiotherapy Evidence Database scale

Supplementary eTable 2 Sensitivity analysis for time to first flatus.

Study	Effect size	95%CI	P	I^2
Li S 2017[21]	-0.98	-1.31, -0.65	P < 0.00001	72%
Toronui T 2020[28]	-0.97	-1.31, -0.64	P < 0.00001	72%
Wang X 2018[29]	-1.03	-1.35, -0.70	P < 0.00001	70%
Wang XQ 2018[30]	-0.99	-1.33, -0.66	P < 0.00001	72%
Yang QH 2012[34]	-0.96	-1.28, -0.63	P < 0.00001	71%
Bai HM 2012[23]	-0.93	-1.25, -0.62	P < 0.00001	69%
Jin CH 2015[25]	-0.95	-1.28, -0.62	P < 0.00001	70%
Li S 2019[26]	-0.90	-1.19, -0.62	P < 0.00001	63%
Wang Y 2017[32]	-1.04	-1.36, -0.72	P < 0.00001	68%
Xi YX 2015[33]	-1.08	-1.34, -0.82	P < 0.00001	52%

CI: confidence intervals

Supplementary eTable 3 Sensitivity analysis for time to first flatus.

Study	Effect size	95%CI	P	I^2
Toronui T 2020[28]	-0.62	-1.41, -0.17	P = 0.12	89%
Jin CH 2015[25]	-0.83	-1.90, 0.24	P = 0.13	93%
Wang Y 2017[32]	-1.24	-2.33, -0.15	P = 0.03	93%
Xi YX 2015[33]	-1.25	-2.33, -0.17	P = 0.02	93%

CI: confidence intervals

Supplementary eTable 4 Assessment of publication bias.

Outcomes	N	Begg' s test	Egger's test
TFF	10	0.089	0.086
TFF more than 72 h	2	1	Not available
TFD	2	1	Not available
TBS	5	0.174	0.215
PONV (6 h)	8	0.216	0.057
PONV (24 h)	10	0.421	0.693
PONV (48 h)	4	0.117	0.230
MTL (6 h)	2	1	Not available
MTL (24h)	4	0.042	0.112
GAS (6 h)	2	1.00	Not available
GAS (24 h)	3	0.117	0.204

N: number of studies; TFF: time to first flatus; TFD: time first to defecation; TFBS: time to first bowel sound; PONV: postoperative nausea and vomiting; MTL: motilin; GAS: gastrin.

Supplementary eTable 5 Evidence quality assessment according to GRADE

electroacupuncture for POI for POI

Patient or population: patients with POI
Settings:
Intervention: electroacupuncture for POI

Outcomes	Illustrative comparative risks* (95% CI)<br>Assumed risk<br>Control	 Corresponding risk<br>Electroacupuncture for POI	Relative effect (95% CI)	No of Participants (studies)	Quality of the evidence (GRADE)	Comments
**TFF**		The mean tff in the intervention groups was **0.98 standard deviations lower** (1.28 to 0.68 lower)		633 (10 studies)	⊕⊕⊝⊝ **low**[1,2]	SMD -0.98 (-1.28 to -0.68)
**TFF more than 72 h**	**Study population**		**OR 0.16** (0.02 to 1.35)	119 (2 studies)	⊕⊝⊝⊝ **very low**[1,3,4]	
	**83 per 1000**	**14 per 1000** (2 to 109)				
	**Moderate**					
	**83 per 1000**	**14 per 1000** (2 to 109)				
**TFD**		The mean tfd in the intervention groups was **1.23 standard deviations lower** (1.59 to 0.88 lower)		148 (2 studies)	⊕⊕⊝⊝ **low**[1,4]	SMD -1.23 (-1.59 to -0.88)
**TBS**		The mean tbs in the intervention groups was **0.98 standard deviations lower** (1.84 to 0.12 lower)		303 (4 studies)	⊕⊝⊝⊝ **very low**[1,4,5]	SMD -0.98 (-1.84 to -0.12)
**PONV - Postoperative 6 h**	**Study population**		**OR 0.42** (0.27 to 0.64)	453 (8 studies)	⊕⊕⊝⊝ **low**[1,2]	
	**491 per 1000**	**289 per 1000** (207 to 382)				
	**Moderate**					
	**434 per 1000**	**244 per 1000** (172 to 329)				
	**Study population**					

**PONV - Postoperative 24 h**	**359 per 1000**	**205 per 1000** (152 to 275)	**OR 0.46** (0.32 to 0.68)	606 (10 studies)	⊕⊕⊖⊖ **low**[1,2]	
	**Moderate**					
	**383 per 1000**	**222 per 1000** (166 to 297)				
**PONV - Postoperative 48 h**	**Study population**		**OR 0.55** (0.2 to 1.51)	234 (4 studies)	⊕⊕⊖⊖ **low**[2,6]	
	**102 per 1000**	**59 per 1000** (22 to 146)				
	**Moderate**					
	**83 per 1000**	**47 per 1000** (18 to 120)				
**MTL - Postoperative 6 h**		The mean mtl - postoperative 6 h in the intervention groups was **0.93 standard deviations lower** (1.36 to 0.51 lower)		120 (2 studies)	⊕⊖⊖⊖ **very low**[1,2,4]	SMD -0.93 (-1.36 to -0.51)
**MTL - Postoperative 24 h**		The mean mtl - postoperative 24 h in the intervention groups was **0.43 standard deviations lower** (0.89 lower to 0.02 higher)		255 (4 studies)	⊕⊕⊖⊖ **low**[1,5]	SMD -0.43 (-0.89 to 0.02)
**GAS - Postoperatie 6 h**		The mean gas - postoperatie 6 h in the intervention groups was **0.2 standard deviations higher** (1.62 lower to 2.01 higher)		120 (2 studies)	⊕⊖⊖⊖ **very low**[1,4,5]	SMD 0.2 (-1.62 to 2.01)
**GAS - Postoperative 24 h**		The mean gas - postoperative 24 h in the intervention groups was **0.63 standard deviations higher** (0.57 lower to 1.84 higher)		195 (3 studies)	⊕⊖⊖⊖ **very low**[1,4,5]	SMD 0.63 (-0.57 to 1.84)
**VIP (postoperatie 24 h)**		The mean vip (postoperatie 24 h) in the intervention groups was **0.12 standard deviations higher** (0.26 lower to 0.5 higher)		135 (2 studies)	⊕⊖⊖⊖ **very low**[1,4,5,6]	SMD 0.12 (-0.26 to 0.5)

**pHi - pneumoperitoneum for 30 min**		The mean phi - pneumoperitoneum for 30 min in the intervention groups was **0.7 standard deviations higher** (0.47 lower to 1.88 higher)	108 (2 studies)	⊕⊖⊖⊖ **very low**[1,4,5]	SMD 0.7 (-0.47 to 1.88)
**pHi - 30 min after the end of pneumoperitoneum**		The mean phi - 30 min after the end of pneumoperitoneum in the intervention groups was **1.15 standard deviations higher** (0.95 lower to 3.24 higher)	108 (2 studies)	⊕⊖⊖⊖ **very low**[1,4,5]	SMD 1.15 (-0.95 to 3.24)
**PgCO2 - pneumoperitoneum for 30 min**		The mean pgco2 - pneumoperitoneum for 30 min in the intervention groups was **0.87 standard deviations lower** (1.26 to 0.47 lower)	108 (2 studies)	⊕⊖⊖⊖ **very low**[1,4,5]	SMD -0.87 (-1.26 to -0.47)
**PgCO2 - 30 min after the end of pneumoperitoneum**		The mean pgco2 - 30 min after the end of pneumoperitoneum in the intervention groups was **1.06 standard deviations lower** (1.46 to 0.65 lower)	108 (2 studies)	⊕⊖⊖⊖ **very low**[1,3,4]	SMD -1.06 (-1.46 to -0.65)

*The basis for the **assumed risk** (e.g. the median control group risk across studies) is provided in footnotes. The **corresponding risk** (and its 95% confidence interval) is based on the assumed risk in the comparison group and the **relative effect** of the intervention (and its 95% CI).

CI: Confidence interval; **OR:** Odds ratio;

GRADE Working Group grades of evidence

High quality: Further research is very unlikely to change our confidence in the estimate of effect.

Moderate quality: Further research is likely to have an important impact on our confidence in the estimate of effect and may change the estimate.

Low quality: Further research is very likely to have an important impact on our confidence in the estimate of effect and is likely to change the estimate.

Very low quality: We are very uncertain about the estimate.

[1] the risk of bias regarding blinding of participants (performance bias) was high

[2] different comparators

[3] large confidence intervals

[4] small sample size

[5] high heterogeneous

[6] No explanation was provided
